# Supplementary material for: prewas: data pre-processing for more informative bacterial GWAS
Source: Microb Genom. 2020 Apr 20;6(5):e000368. doi: 10.1099/mgen.0.000368 (PMC7371116; doi:10.1099/mgen.0.000368)
Supplement: Supplementary material 1 [file mgen-6-368-s001.pdf]

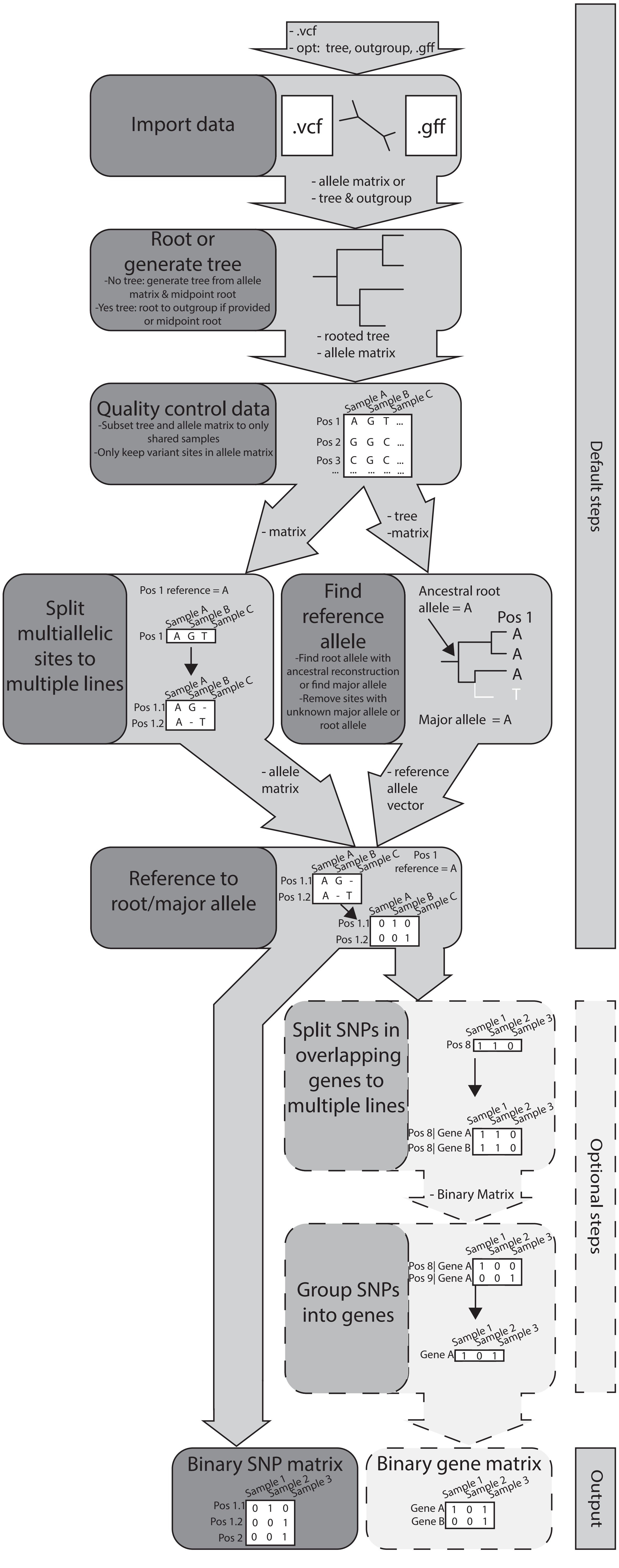

Figure S1. Detailed prewas workflow.

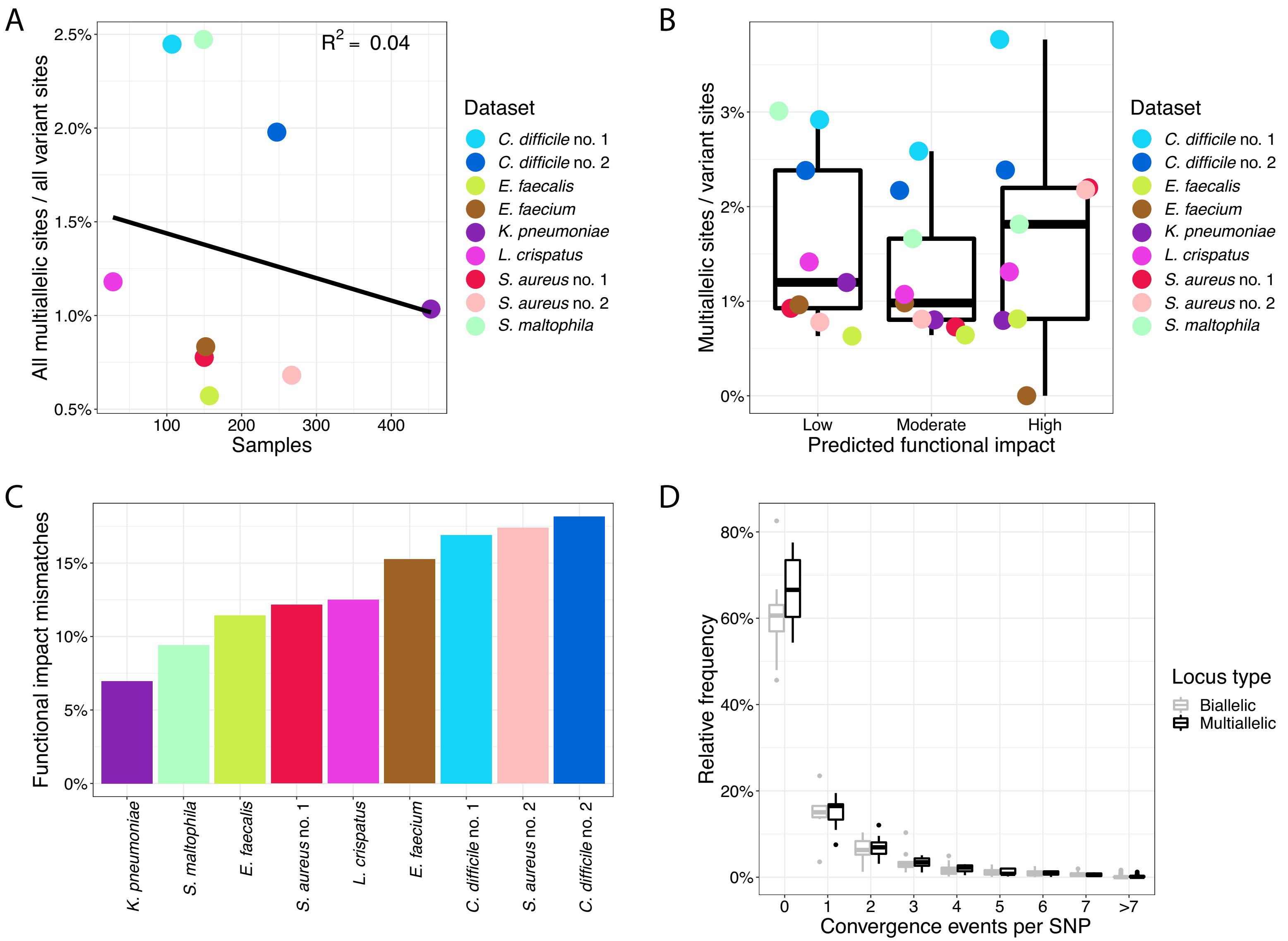

Figure S2. Multiallelic Sites.

(A) Independence observed between sample size and prevalence of multiallelic sites. (B) Prevalence of multiallelic sites compared to variant sites with each subset to the various predicted functional impacts. Any multiallelic site with specific impact is compared to any variant site with the same predicted impact. (C) Multiallelic sites with discordant predicted functional impact among alternative alleles. (D) The relative frequency of the number of times an allele arises on the tree. At multiallelic sites, all minor alleles are treated separately.

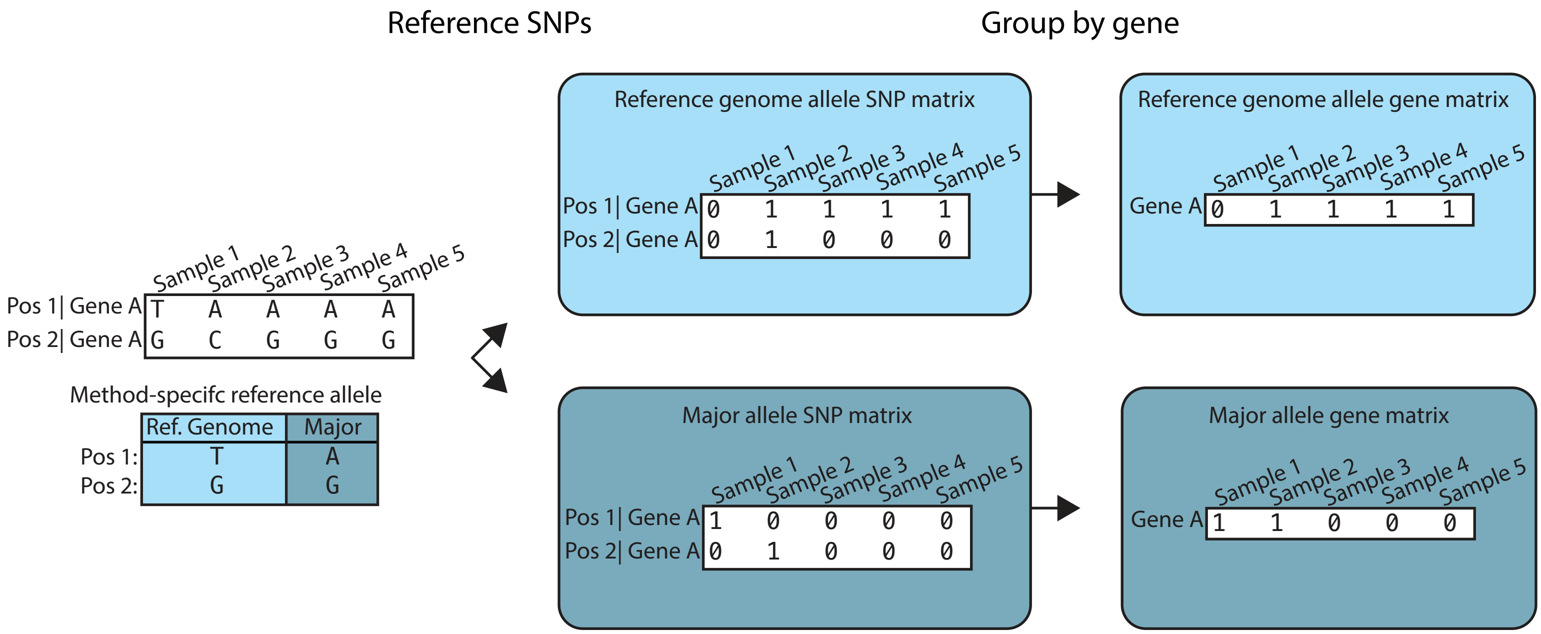

Figure S3. Masking variation at the gene level when grouping into genes.
When not confident in the ancestral reconstruction or ancestral reconstruction is not computationally feasible, we suggest referencing to the major allele. In this example, referencing to the reference genome allele masks variation at the gene level. When referencing to the reference genome allele, the variation in Position 2 gets masked by the variation in Position 1 when grouped by gene, leading to a likely lack of association. However, if instead we reference to the major allele, the variation in Gene A is maintained, allowing for potential associations to be detected.

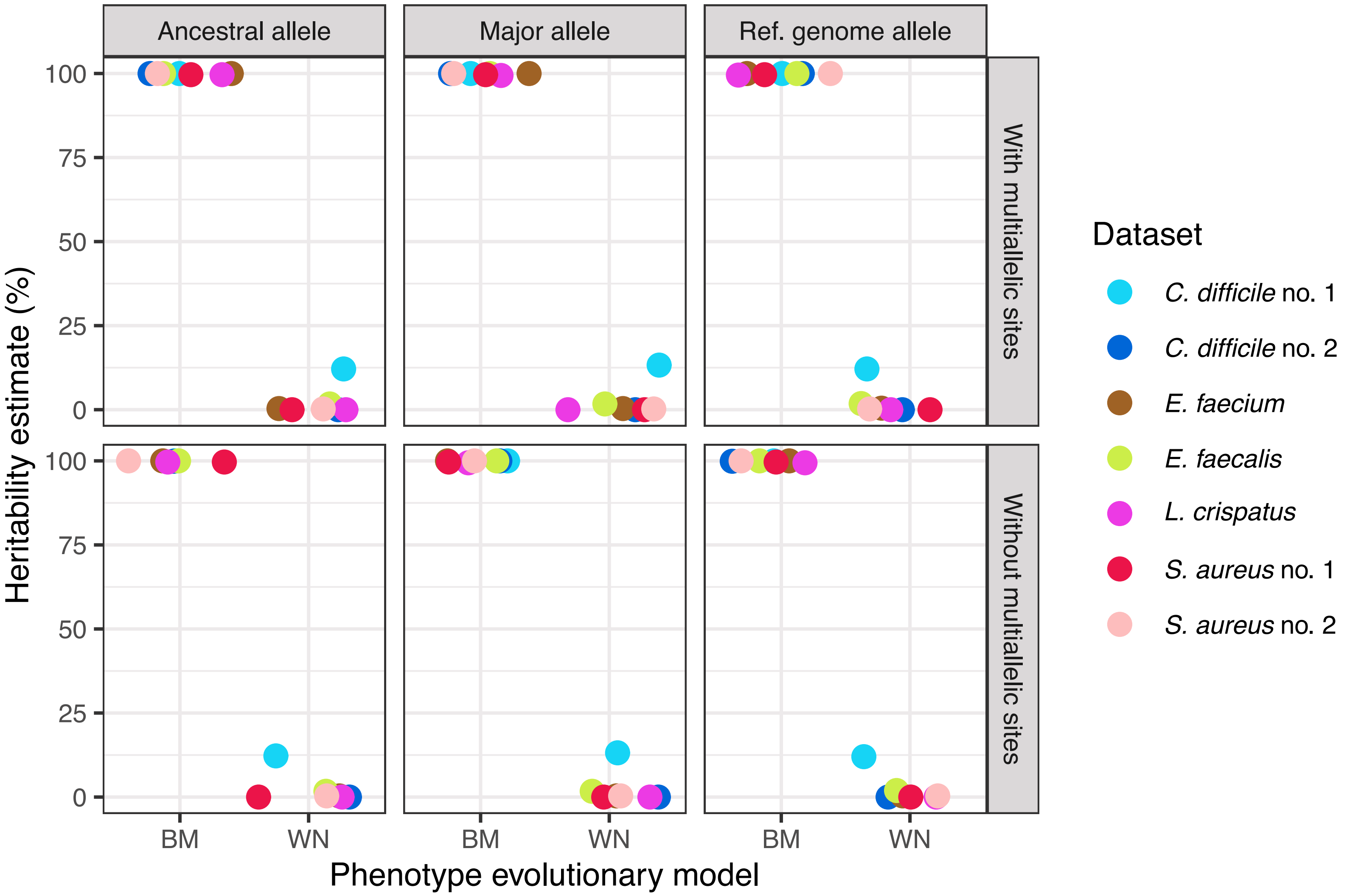

Figure S4. Heritability with different sets of reference alleles, with and without multiallelic sites. Heritability of a simulated normally distributed phenotype with three different reference allele options (ancestral allele, major allele, and reference genome allele), with and without multiallelic sites, for each dataset. BM=Brownian motion, WN=white noise.

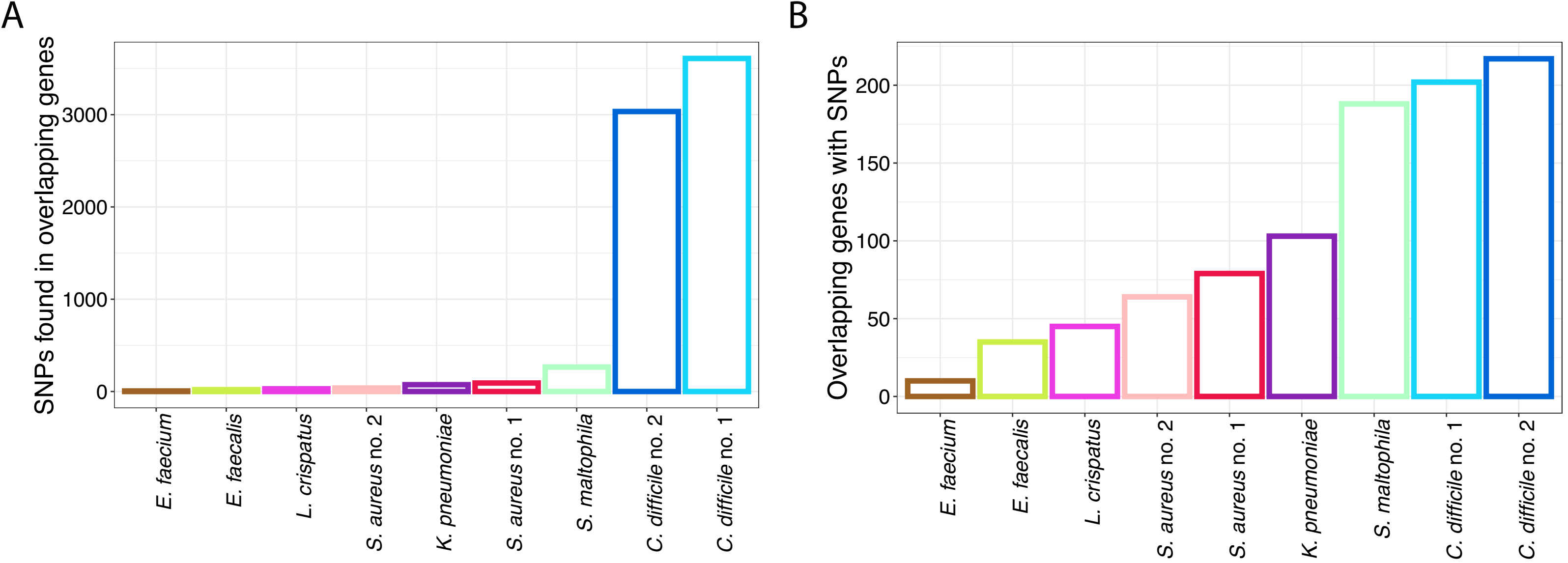

Figure S5. Overlapping genes with SNPs.
(A) SNP loci found in positions shared by overlapping genes. (B) Overlapping genes with SNPs found in the overlapping positions.

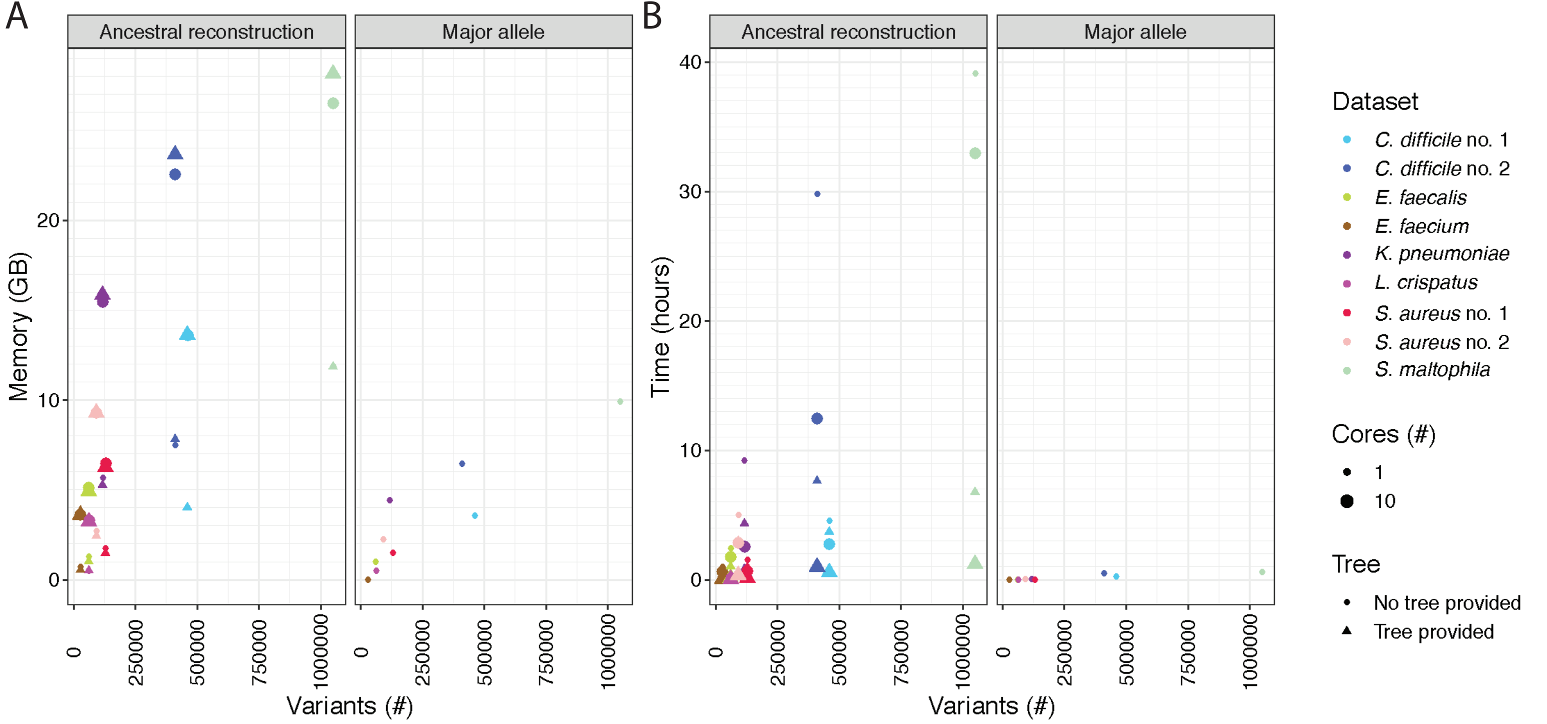

Figure S6. Resource Utilization.
(A) Memory usage. (B) Run time.
